# A New Quantitative Cell-Based Assay Reveals Unexpected Microtubule Stabilizing Activity of Certain Kinase Inhibitors, Clinically Approved or in the Process of Approval

**DOI:** 10.3389/fphar.2020.00543

**Published:** 2020-04-30

**Authors:** Sacnicte Ramirez-Rios, Sophie Michallet, Leticia Peris, Caroline Barette, Clotilde Rabat, Yangbo Feng, Marie-Odile Fauvarque, Annie Andrieux, Karin Sadoul, Laurence Lafanechère

**Affiliations:** ^1^Institute for Advanced Biosciences, Team Regulation and Pharmacology of the Cytoskeleton, INSERM U1209, CNRS UMR5309, Université Grenoble Alpes, Grenoble, France; ^2^Grenoble Institute of Neurosciences, INSERM U1216, Université Grenoble Alpes, CEA, Grenoble, France; ^3^Univ. Grenoble Alpes, CEA, INSERM, IRIG, BGE, Genetics and Chemogenomics, Grenoble, France; ^4^Reaction Biology Corporation, Malvern, PA, United States

**Keywords:** cell-based assay, microtubule stabilization, kinase inhibitors, polypharmacology, microtubule dynamics

## Abstract

Agents able to modify microtubule dynamics are important anticancer drugs. The absence of microtubules resulting from drug-induced depolymerization is easy to detect. However the detection of a stabilized microtubule network needs specific assays since there is not a significant visual difference between normal and stabilized microtubule networks. Here, we describe a quantitative cell-based assay, suitable for automation, which allows the detection of stabilized microtubules without the need of microscopic examination. The rationale of this assay is based on the drug-induced resistance of the microtubule network to the depolymerizing agent combretastatin A4 and the subsequent detection of the residual microtubules by immunoluminescence. Using this assay to screen a kinase inhibitor library allowed the selection of seven known kinase inhibitors: selonsertib, masatinib, intedanib, PF0477736, SNS-314 mesylate, MPI0479605, and ponatinib. The yet undescribed ability of these inhibitors to stabilize cellular microtubules was confirmed using additional markers of stable microtubules and time-lapse video-microscopy to track individual microtubules in living cells. None of the compounds interacted, however, directly with tubulin. By employing other inhibitors of the same kinases, which have structurally unrelated scaffolds, we determined if the microtubule stabilizing effect was due to the inhibition of the targeted kinase, or to an off-target effect. Many of these inhibitors are clinically approved or currently assayed in phase 2 or phase 3 clinical trials. Their microtubule-stabilizing effect may account for their therapeutic effect as well as for some of their adverse side effects. These results indicate also a possible repurposing of some of these drugs.

## Introduction

Pharmacological treatments used in cancer chemotherapy generally fall into two main classes: cytotoxic drugs and targeted therapies (Winkler et al., 2014). Among drugs targeting specific pathways, kinase inhibitors have played an increasingly prominent role. Currently, more than 25 oncology drugs that target kinases have been approved, and several additional kinase inhibitors are in various stages of clinical evaluation. However, such drugs often serendipitously display widely varying target profiles beyond their intended targets. While these “off’” targets are often either unknown or disregarded, they confer an inherent potential for polypharmacology applications. Notably, phenotypic screening approaches have found that some kinase inhibitors show antitumor activity unrelated to inhibition of their cognate targets, for which the underlying mechanism of action is therefore not apparent but likely involves one or more non-canonical targets (Remsing Rix et al., 2014).

Among cytotoxic drugs, microtubule-targeting agents belong to one of the oldest and most diverse family of anticancer agents. Microtubules (MTs) are cylindrical polymers of α- and β-tubulin heterodimers. These polymers are highly dynamic and their polymerization dynamics is tightly regulated through the binding of microtubule-associated proteins (MAPs). Both MAPs and tubulin are further subjected to regulatory post-translational modifications. Microtubule-targeting agents act primarily by altering MT dynamics. They are roughly classified into microtubule-stabilizing agents, such as taxanes or epothilones, and microtubule-destabilizing agents, such as vinca alkaloids, combretastatin, and colchicine. The vinca alkaloids bind to a site of β-tubulin located at the interface between two tubulin dimers (Gigant et al., 2005), whereas colchicine binds to a site of β-tubulin located at the intra-dimer interface, between α- and β-subunits (Ravelli et al., 2004). More recently, it has been shown that the combretastatin binding-site is the same as the colchicine binding-site (Zhou et al., 2016). Although taxanes and vinca alkaloids are powerful anticancer drugs, they also have severe side effects such as myelosuppression or neurotoxicity. Moreover, many cancers are or become resistant to these drugs (Dumontet and Jordan, 2010). This resistance often results from the overexpression of βIII–tubulin [an isoform of β-tubulin (Kavallaris, 2010)] and ATP-binding cassette transporters (ABC transporters), which function as drug-efflux pumps (Haimeur et al., 2004). A strategy to circumvent these limitations is to identify new chemical scaffolds that are not substrates of resistance mechanisms or drugs that target regulators of MT-dynamics rather than compounds interacting directly with the MT network. The selection of such drugs can be achieved by phenotypic screening strategies using cell-based assays that probe MT dynamics (Mayer et al., 1999; Roberge et al., 2000; Vassal et al., 2006; Prudent et al., 2012; Prudent et al., 2013; Martinez et al., 2015). High-content phenotypic screening, based on microscopic analysis of the cellular MT cytoskeleton or on the cell morphology can easily detect an agent that depolymerizes MTs (Ross-Macdonald et al., 2008; Munoz, 2017; Hoque et al., 2018). Distinguishing a stabilized MT network based on its morphology is generally delicate, except for the stabilization by paclitaxel (PTX), which at high doses induces the formation of characteristic MT bundles. Assays based on the detection of indirect markers of stabilized MTs, such as the immunodetection of post-translationally modified MTs (i.e., detyrosinated or acetylated MTs) (Vassal et al., 2006; Hammond et al., 2008; Peris et al., 2009), are thus often required. Here, we describe a new sensitive and quantitative cell-based assay, suitable for scaling up for high throughput screening (HTS), which allows the identification of microtubule-stabilizing agents without the need of microscopic image analysis. We used this assay to screen a chemical library of kinase inhibitors. We have chosen to focus on kinase inhibitors with two goals. First, we aim at identifying kinases regulating directly or indirectly the tubulin network. Second, kinase inhibitors are now commonly used in chemotherapy but could be suspected to possess off-targets that may well be involved in the stabilization of the microtubule network known to efficiently prevent cancerous cell proliferation. Tubulin itself could be an off-target of some kinase inhibitors. Indeed, structural analyzes show that the binding site of taxanes, like that of colchicine, is mainly hydrophobic in nature, large in size and fairly deep (Steinmetz and Prota, 2018). These characteristics are similar to those of the catalytic site of kinases. Accordingly, it has been shown that several kinase inhibitors can bind to the colchicine site of tubulin and inhibit its polymerization (Bohnacker et al., 2017; Tanabe, 2017). Conversely, nocodazole, a drug known to inhibit the polymerization of tubulin is a high-affinity ligand of the kinases ABL, c-KIT, BRAF, and MEK (Park et al., 2012).

This approach led us to discover that some of those inhibitors, which are already in clinics or currently assayed in phase 2 or phase 3 clinical trials, have potent and unknown cell MT stabilizing properties, without targeting tubulin directly. Their newly discovered ability to stabilize MTs is likely contributing to the cytotoxicity of these drugs and may be responsible for adverse side effects.

## Materials and Methods

### Cells and Reagents

Human HeLa cells were obtained from the American Type Culture Collection and cultured at 37°C in 5% CO_2_ in the recommended media supplemented with 10% fetal bovine serum (51810-500, Dutscher). The SYNlibrary Kinome Kinase Inhibitor Library, containing 187 kinase inhibitors, was purchased from SYNkinase (Australia) in a ready-to-screen format (2 µl at 2 mM in 100% DMSO, one sample per compound). The following molecules were further purchased and prepared at a 10 mM stock solution in DMSO, aliquoted and stored at −20°C; Masatinib, Selonsertib hydrochloride, Intedanib, and PF-0477736 were from COGER. MPI 0479605, SNS-314 mesylate, and Ponatinib were from Selleckchem. Paclitaxel, nocodazole, and combretastatin A4 were from Sigma. GST-cofilin was prepared as previously described (Martinez et al., 2015) and purified brain bovine tubulin was purchased at the Centro de Investigaciones Biológicas, CSIC, Madrid, Spain.

SR series LIMK inhibitors: SR10847 (1-(2-aminoethyl)-1-(4-chlorophenyl)-3-(4-(5-methyl-7H-pyrrolo[2,3-d]pyrimidin-4-yl)phenyl)urea), SR10854 (1-(2-aminoethyl)-1-(4-methoxyphenyl)-3-(4-(5-methyl-7H-pyrrolo[2,3-d]pyrimidin-4-yl)phenyl)urea), SR10905(1-(2-aminoethyl)-1-(4-chlorophenyl)-3-(2-fluoro-4-(5-methyl-7H-pyrrolo[2,3-d]pyrimidin-4-yl)phenyl)urea) were synthesized and characterized using similar procedures as described in Yin et al. (2015). In a cell-free LIMK1 biochemical assay, the IC50 values of the three compounds are 21, 27, and 15 nM, respectively.

### Antibodies

The primary antibodies used were anti-α-tubulin [clone α3A1 (Peris et al., 2006)], anti-detyrosinated tubulin [L4 (Peris et al., 2006)], anti-cofilin (Cell Signaling, 5175), anti-phosphocofilin (ser3) (Cell Signaling, 3313). For immunofluorescence assays secondary antibodies were coupled to Alexa 488 (Jackson Immuno-Research Laboratories, 115-545-003) or Cy3 (Jackson Immuno-Research Laboratories, 111-165-003). For Western blots, secondary antibodies coupled to HRP were used (Jackson Immuno-Research Laboratories, 711-039-152).

### HTS Cell-Based Assay and Procedure for the Library Screening

Seven thousand five hundred HeLa cells were seeded in 96-well microplates (Greiner #655083) in 100 µl of complete medium per well and then incubated at 37°C in 5% CO_2_. Twenty-four hours after seeding, cells were treated with compounds (one compound per well) at 10 µM final concentration for 90 min, Pyr1 at 25 µM, 0.25% DMSO and DMSO alone at 0.25% were used as positive and negative controls (eight wells per microplate and per control), respectively. Then combretastatin A4 was added to each well at 0.5 µM final concentration for 30 min. After medium aspiration, treated cells were permeabilized for 10 min using 100 µl per well of OPT buffer (80 mM Pipes, 1 mM EGTA, 1 mM MgCl_2_, 0.5% Triton X-100, and 10% glycerol, pH 6.8) pre-warmed to 37°C. After buffer aspiration, cells were fixed overnight at room temperature using 100 µl per well of 4% formaldehyde in PBS pH 7.2. Cells were washed three times in PBS pH 7.2, 0.1% Tween-20 (150 µl per well), then 50 µl of α3A1 anti-tubulin antibody (1/5,000 in PBS pH 7.4, 0.3% BSA, 0.03% NaN3) were added for 45 min. After washing of cells as described above, 50 µl of anti-mouse antibody coupled to HRP (1/2,000 in PBS pH 7.4, 0.3% BSA, 0.03% NaN3) were added for 45 min. Then cells were washed again and, 50 µl of ECL Western blotting substrate (Pierce #32106) were added to each well and the luminescent signal was read after 5 min of incubation.

For the robotic execution of the assay at the HTS facility, the pipetting, washing, and reading steps were performed using MCA96 pipetting arm, HydroSpeed™ plate washer and Infinite^®^ M1000 plate reader (Tecan, Switzerland), respectively. The Z’ factor was calculated according to Zhang et al. (1999).

Because chemiluminescence is typically about two orders of magnitude more sensitive than fluorescence, we have chosen this type of detection. The alternative use of a fluorescent secondary antibody is also possible (Vassal et al., 2006).

### Immunofluorescence

Cells were fixed and processed for immunofluorescence analysis of the MT network as previously described (Prudent et al., 2012).

### Western Blots

HeLa cells were treated with various concentrations of the kinase inhibitors for 2 h or 48 h, as indicated. DMSO was used as control. After the treatment, cells were washed with cold PBS and then scraped in RIPA buffer (50 mM Tris-HCl pH 7.4, 150 mM NaCl, 1% Triton X-100, 0.1% SDS, 20 mM *p*-glycerol phosphate, 1 mM sodium vanadate, 10 mM sodium fluoride, and protease inhibitor cocktail [Roche]). Samples were sonicated and centrifugated (20 min, 15,000 rpm, 4°C) and protein concentration was quantified using the Lowry method (Biorad). Equal amounts (5–10 µg) of proteins were loaded onto 4–20% gradient polyacrylamide gels (Biorad) and separated by electrophoresis. Proteins were transferred to PVDF membranes and probed with antibodies as previously described (Prudent et al., 2012). The ECL Plus Chemiluminescent Kit (Biorad) was used for detection.

### *In Vitro* Microtubule Assembly Assay

Pure tubulin was equilibrated in 80 mM PIPES, 1 mM EGTA, 1 mM MgCl_2_, pH 6.8 buffer (BRB80) and centrifuged at 75,000 rpm for 10 min in an TLA-100 rotor in a Beckman Optima TLX centrifuge. The tubulin concentration was measured spectrophotometrically at 275 nm (ε = 107.000 M^-1^ cm^-1^) and then diluted to 10 μM. The samples were supplemented with 1 mM GTP and the desired drug at 10 μM (or the vehicle, i.e. DMSO). The time course of assembly at 37°C was detected as turbidity by measuring the absorbance of the samples at 350 nm using a VISIONlite spectrophotometer (Thermo scientific).

### Microtubule Sedimentation Assay

In a first step, MTs were polymerized for 30 min at 35°C using 60 µM of tubulin in BRB80 buffer with 1 mM of GTP in a total volume of 20 µl. After MT polymerization, the different kinase inhibitors were added at 10 µM final concentration and again incubated for 30 min at 35°C. MTs were then subjected to depolymerization by cold treatment at 4°C for 30 min and carefully loaded on 40 µl cushion of 60% (w/v) sucrose in BRB80 buffer before centrifugation at 70,000 rpm (Beckman rotor TLA-100) for 45 min at 35°C. After centrifugation, pellets were dissolved in an equal volume as supernatants and proteins were analyzed by SDS-PAGE and revealed by Coomassie blue stain.

### Kinase Assay

*In vitro* phosphorylation assay of GST-cofilin by LIMK1 was carried out as described (Martinez et al., 2015). Briefly, 2.9 µM of GST-cofilin was incubated for 10 min at 30°C in the presence of 100 nM human LIMK1 (Millipore #14-656) and 10 µM of different kinase inhibitors in 50 mM MOPs pH 7.0, 200 µM EDTA, 25 mM Mg(OAc)2, 1 mM DTT, and 200 µM ATP, in a final volume of 30 µl. Kinase reactions were stopped by the addition of SDS sample buffer, boiled, and then subjected to SDS-PAGE and Western blotting.

### Lentivirus Production and Cell Infection

The mRuby3 construct that binds MTs +TIPS was a generous gift of Marina Mikhaylova’s lab (Honnappa et al., 2009). Briefly, a short peptide motif, Ser-x-Ile-Pro (SxIP), of the EB1 interacting protein MACF2 was tagged with mRuby3 and cloned at the place of mCherry into a pLV-mCherry Plasmid (#36084, Addgene). 1 × 10^7^ HeLa cells were infected with 10^8^ IU of mRuby3-MT+tips lentivirus and maintained in RPMI supplemented with 10% FBS. After amplification, half of them were frozen and the rest was used for experiments. Every two weeks, new cells were thawed to perform the experiments.

### Measurements of Microtubule Dynamics Using Videomicroscopy

Fluorescence imaging of mRuby3-MT+tips of MTs in live HeLa cells maintained at 37°C, 5% CO_2_ was performed using an inverted microscope (Axio Observer, Zeiss) coupled to a spinning-disk confocal system (CSU-W1-T3, Yokogawa) connected to a wide-field electron-multiplying CCD camera (ProEM+ 1024, Princeton Instrument). The microscope setup was controlled by MetaMorph software. Time-lapse microscopy imaging was performed after 2 h of treatment with 0.2% DMSO (control) or drugs at a concentration of 10–20 µM. Acquisitions were performed every 1.5 sec with a 100 msec exposure time during 2 min using a 63× NA 1.46 oil-immersion objective. Kymographs of MT plus end dynamics were done using ImageJ software and a homemade KymoTool (Ramirez-Rios et al., 2016). For quantification of plus end dynamics the time-lapse movies were visually inspected and only mRuby3-MT+tip comets that were visible for at least three consecutive frames were included in the analysis. The velocities of mRuby3-MT+tip comets were calculated by dividing the distance travelled by the time spent travelling. The average length of MT growth events was also measured. The number of comets within a cell was quantified in the first image of each stack, by measuring the number of comets using “find maxima” in image J and dividing by the cell surface.

## Results

### Establishing a Cell-Based Assay for the Detection of Microtubule Stabilizing Agents

The assay probes a drug-induced resistance of the MT network to a depolymerization provoked by agents such as combretastatin A4 (CA4) or nocodazole. CA4 or nocodazole binds free tubulin dimers and prevents their incorporation into MTs, leading to a progressive loss of the polymerized MT network (Florian and Michison, 2016). Stabilized MTs with slow dynamics have reduced exchanges of their tubulin content with the free tubulin pool, and are thus less sensitive to nocodazole- or CA4- induced depolymerization (Saoudi et al., 1998; Prudent et al., 2012). Nocodazole appears, however, to be less tubulin specific as it has been reported to be a high affinity ligand for cancer-related kinases including Abl, cKit, BRAF, and MEK (Park et al., 2012). We thus decided to choose CA4 as MT depolymerizing agent to establish the assay.

**Figure 1:**
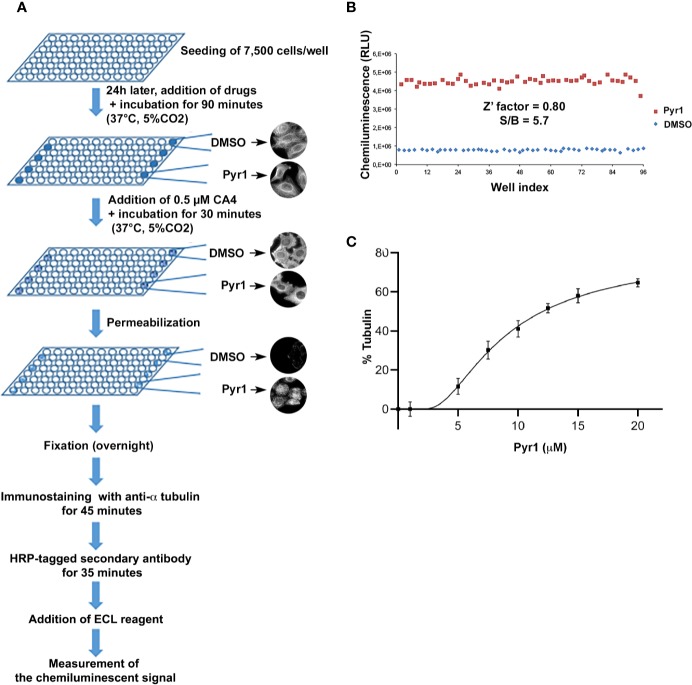
A cell-based assay for detecting microtubule stabilizing agents. **(A)** Schematic representation of the different steps of the assay. **(B)** Evaluation of the intra-assay repeatability. HeLa cells were seeded on a microplate. After 24 h, they were treated for 90 min with DMSO (negative control, blue diamonds) or Pyr1 25 µM (positive control, red squares). Then CA4 was added to each well at a final concentration of 0.5 µM for 30 min. Cells were then processed for chemiluminescence, as described in the *Materials and Methods* section. Each point represents the luminescence intensity (expressed as Relative Luminescence Units) obtained for each well of the 96-well microplate. **(C)** Evaluation of the assay sensitivity. HeLa cells were seeded on microplates. After 24 h, they were treated for 90 min with DMSO (negative control) or with different concentrations of Pyr1 as indicated. CA4 was added to each well at a final concentration of 0.5 µM for 30 min. Cells were then processed for chemiluminescence, as described in the *Materials and Methods* section. Results are expressed as % of MTs resistant to CA4 induced depolymerization, with 100% corresponding to cells treated with DMSO without CA4 and 0% corresponding to cells treated with DMSO and CA4; mean +/− SEM.

The main steps of the assay are illustrated in **Figure 1A** and described in the *Methods* section.

To set up the assay for HTS, a positive control—i.e. a known stabilizing agent—and a control with no activity were defined. Because the PTX stabilizing activity is uniquely high we decided to use a less potent positive control, to better adapt the dynamic window of the assay and to increase its sensitivity. We thus used Pyr1, which induces a partial stabilization of cellular MTs by LIM kinase (LIMK) inhibition (Prudent et al., 2012), as positive control for a microtubule stabilizing agent. Using Pyr1 and the vehicle DMSO, as positive and negative control respectively, we set up a high quality, reproducible assay as assessed by calculating signal-to-background ratios and Z**′** factor according to Zhang and others (Zhang et al., 1999). We obtained a Pyr1/DMSO ratio of 5.7 and a Z’ value of 0.80, indicating that these assay conditions are excellent for HTS (**Figure 1B**). To evaluate the sensitivity of the assay we tested the effect of different concentrations of Pyr1 on the luminescence intensity measured by the microplate reader. As shown in **Figure 1C**, a dose-dependent MT stabilization could be detected for Pyr1. These results demonstrate that the assay is statistically robust, quantitative, and sensitive enough for HTS

### Identifying Microtubule-Stabilizing Compounds in a Library of Kinase Inhibitors

**Figure 2:**
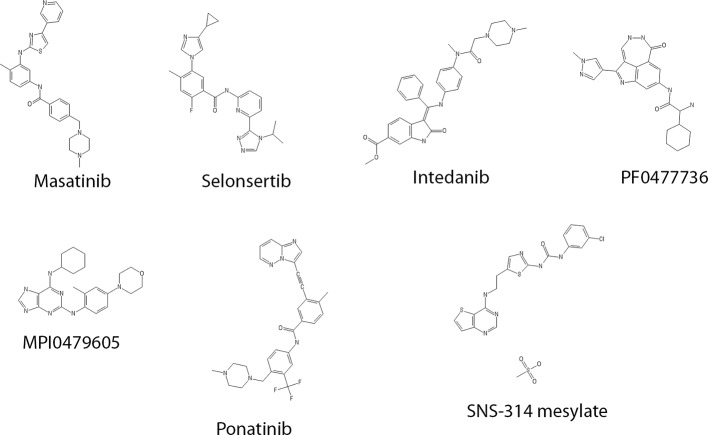
Structure of the selected kinase inhibitors.

We performed a pilot screen of a commercially available chemical library of 187 known kinase inhibitors. In addition, the library was supplemented with the known microtubule stabilizing agents, PTX and epothilone (Steinmetz and Prota, 2018). We also added a collection of 12 bis-aryl urea derivatives for which a LIM kinase (LIMK) inhibitory activity has been previously described *in vitro* in a cell-free system (Yin et al., 2015). Compounds were screened at a final concentration of 10 µM. Compounds that showed an activity superior to 50% of Pyr1 activity were considered as hits. **Table 1** shows that different known kinase inhibitors—masatinib, selonsertib, intedanib, PF0477736, SNS-314 mesylate, MPI0479605, and ponatinib—were found positive using this screening assay. Regarding compound structures (**Figure 2**), except for the fairly common N-methylpiperazine moiety (present in intedanib, masatinib, and ponatinib), no recurrent patterns are observed.

**Table 1:** Hits selected by screening.

Compound	Target	% Pyr1-like activity
Paclitaxel	Tubulin	265
Epothilone B	Tubulin	253
Masatinib	Tyrosine kinase inhibitor	141
Selonsertib	Apoptosis signal-regulating kinase 1 (ASK1)	205
Intedanib	Receptor tyrosine kinase (RTK)	165
PF0477736	Chk1/Chk2	112
SNS-314 mesylate	Aurora kinases A, B, and C	107
MPI0479605	Monopolar spindle 1 (Mps1) kinase	114
Ponatinib	Receptor tyrosine kinase (RTK)	73
SR10905	LIM Kinase (Yin et al., 2015)	82
SR10854	LIM Kinase	147
SR10847	LIM Kinase	99

PTX and epothilone B, known MT stabilizers, were the most active compounds. Several compounds (SR10905, SR10854, and SR10847) from the added bis-aryl urea derivatives with LIMK inhibitory activities were also detected as active compounds, revealing that they were cell permeant and able to induce a stabilization of MTs, as it has been described for the LIMK inhibitor Pyr1 (Prudent et al., 2012). These results indicate that the assay is indeed able to select cell-permeant MT stabilizing agents.

### Comparative Analysis of the Microtubule Stabilizing Activity of the Selected Kinase Inhibitors

**Figure 3:**
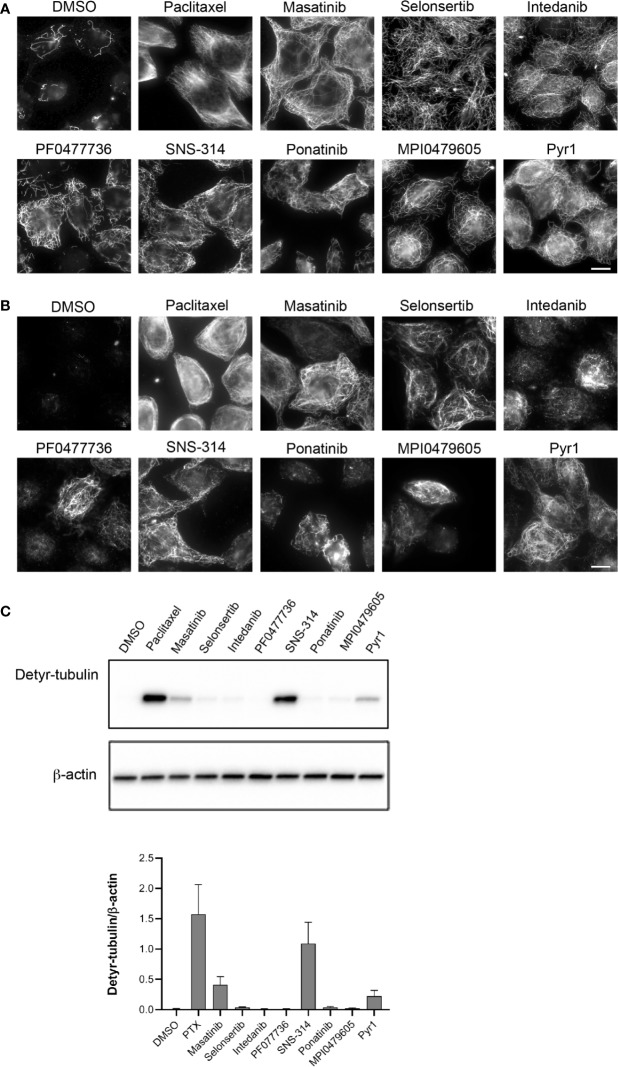
Confirmation of potent microtubule stabilization after treatment of cells with the selected protein kinase inhibitors. **(A)** Kinase inhibitors protect MTs from CA4-induced depolymerization. HeLa cells were incubated for 90 min with 0.25% DMSO, 10 µM paclitaxel, or 10 µM of the indicated kinase inhibitors. CA4 (0.5 µM) was then added for 30 min. Cells were permeabilized and processed for immunofluorescence using an anti-α-tubulin antibody. Scale bar, 10 µm. **(B)** Treatment with the kinase inhibitors increases the detyrosinated MT content of cells. HeLa cells were treated for 2 h with 0.25% DMSO, 10 µM paclitaxel, or 20 µM of the indicated kinase inhibitors. Cells were then permeabilized and processed for immunofluorescence, using an antibody against detyrosinated tubulin. Scale bar, 10 µm. **(C)** Western blot to quantify the effect of kinase inhibitors on the tubulin detyrosination status. HeLa cells were treated for 2 h with 0.25% DMSO, 10 µM paclitaxel, or 20 µM of the indicated kinase inhibitors. Cellular protein extracts were separated by SDS-PAGE and Western blotted to detect detyrosinated tubulin (Detyr-tubulin) and β-actin. A representative Western blot of three independent experiments is shown. The intensity of the bands was quantified using ImageJ software and the calculated ratios are shown as mean +/− SEM on the lower panel.

The original screen was performed with only one data point and with molecules already dissolved and stored in microplate wells. These results needed thus to be confirmed with a new batch of freshly dissolved pure compounds. We first confirmed the results of the screen by microscopic examination. As expected, immunofluorescence of the cellular MT network showed that MTs are resistant to CA4-induced depolymerization after treatment with these compounds (**Figure 3A**).

Stable MTs are specific substrates of some enzymes responsible for tubulin post-translational modifications (Lafanechere and Job, 2000). For instance, the protein complex VASH/SVBP, catalyzing the detyrosination of tubulin, acts preferentially on MTs rather than on the free tubulin dimer (Aillaud et al., 2017). Immunofluorescence (**Figure 3B**) showed that the amount of detyrosinated MTs was indeed clearly increased in cells treated with SNS-314 mesylate, masatinib, and selonsertib, and moderately increased in cells treated with intedanib and ponatinib, when assayed at a concentration of 20 µM. A few scattered cells with detyrosinated MTs were found in the PF0477736 and MPI0479605 treated cell populations. As noticed in previous studies (Prudent et al., 2012; Martinez et al., 2015) the generation of detyrosinated MTs after treatment with MT stabilizing drugs was somehow heterogeneous among cells. The analysis of the detyrosination level of tubulin in whole cell lysates using Western blot confirmed the important detyrosination induced by SNS-314 mesylate and masatinib treatment (**Figure 3C**).

**Figure 4:**
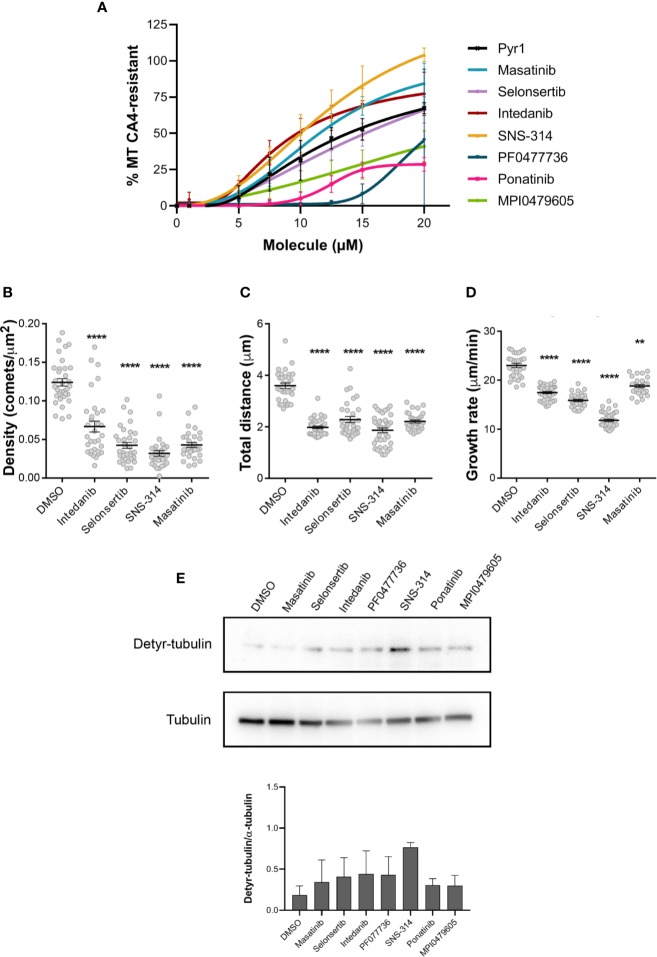
Comparative analysis of the effect of the selected kinase inhibitors on microtubule dynamics in HeLa cells. **(A)** Comparison of the MT stabilizing effect of the different kinase inhibitors. Different doses of the kinase inhibitors were applied to HeLa cells in microplates and their MT stabilizing effect was assessed after a 30 min CA4 (0.5 µM) treatment, using the luminescent assay, as described in the *Materials and Methods* section. Pyr1 was used as positive control. Results are expressed as % of MTs resistant to CA4 induced depolymerization, with 100% corresponding to cells treated with DMSO without CA4 and 0% corresponding to cells treated with DMSO and CA4; mean +/− SEM. **(B–D)** Analyses of the dynamic instability parameters of MTs in HeLa cells treated with the different kinase inhibitors. The analyses were performed on the time-lapse movies (**Supplementary Movie 1**) as described in *Materials and Methods*, using DMSO as control. Results are expressed as mean +/− SEM of three independent experiments. The samples were compared to DMSO using Kruskal-Wallis test with Dunn’s multi comparison test. **: p < 0.01; ****: p < 0.0001. **(E)** Effect of longer exposition of cells to inhibitors on tubulin detyrosination. HeLa cells were treated for 48 h with 0.25% DMSO or 100 nM of the indicated kinase inhibitors. Cellular protein extracts were separated by SDS-PAGE and Western blotted to detect detyrosinated tubulin (Detyr-tubulin) and α-tubulin. A representative Western blot of three independent experiments is shown. The intensity of the bands was quantified using ImageJ software and the calculated ratios are indicated as mean +/− SEM in the lower panel.

Then, in an attempt to rank these compounds, we tested the MT stabilizing effect of different concentrations of these compounds, using the above-described quantitative luminescence assay. As shown in **Figure 4A**, SNS-314 mesylate, masatinib, and intedanib were found more active than Pyr1. Selonsertib was as active as Pyr1, whereas MPI0479605, ponatinib, and PF0477736 showed a lower stabilizing activity.

Finally, we also analyzed the effect of the four most potent compounds—i.e. selonsertib, intedanib, masatinib, and SNS-314 mesylate—on MT dynamics, by following the fast growing plus ends of MTs using time-lapse fluorescence microscopy. To this end, we infected HeLa cells with a Ruby-construct that presents the Ser-x-Ile-Pro (SxIP) motif, which binds to the endogenous MT plus-end binding protein EB1 (Honnappa et al., 2009) (**Supplementary Movie 1**). In a preliminary experiment, we found that masatinib has the most potent effect on MT dynamics and its concentration had to be lowered to 10 µM to measure dynamic instability parameters, as a 20 µM concentration completely hampered microtubule dynamics. We observed for all these compounds a decrease of the number of comets per area (**Figure 4B**), indicative of a stabilization of the MTs (Prudent et al., 2012). In addition, the total distance traveled by the plus ends during the observation period is decreased (**Figure 4C**) as well as their rate of growth (**Figure 4D**), indicating that these kinase inhibitors are able to slow down MT dynamics.

To test if a longer exposure of the cells to lower doses of compounds could also induce a stabilization of the MTs, we exposed the cells for 48 h to a 100 nM concentration of the compounds and then examined the content in detyrosinated tubulin, as an indicator of MT stabilization. As shown on **Figure 4E**, all the compounds except masatinib were able to generate detyrosinated tubulin, indicating that a prolonged incubation of cells with nanomolar doses of these compounds can lead to MT stabilization.

### Cellular Microtubule Stabilization Does Not Result From a Direct Interaction of the Compounds With Tubulin

The MT stabilizing activity of these compounds has never been described, to our knowledge. MT stabilization can result from the direct binding of the compounds to tubulin. Otherwise it can be the consequence of the inhibition of kinases, either the originally targeted kinase or a secondary targeted kinase. It can also result from the binding of the compound to another, yet un-described target.

**Figure 5:**
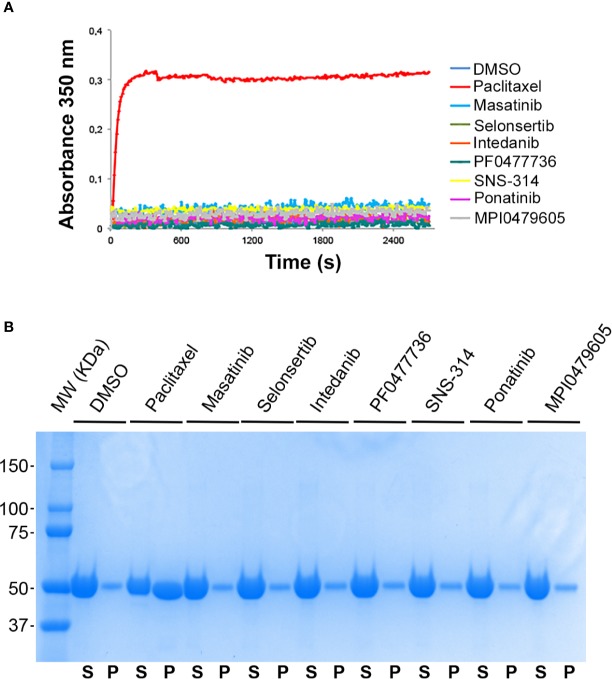
Effect of the kinase inhibitors on microtubule assembly *in vitro*. **(A)** Kinase inhibitors do not promote tubulin polymerization. Time course of polymerization of purified tubulin (10 µM in BRB80 buffer with 1 mM GTP, 37°C) in the presence of vehicle (DMSO, blue line), paclitaxel (10 µM, red line), and the different kinase inhibitors (10 µM), as indicated, measured by turbidimetry at 350 nm. **(B)** Kinase inhibitors do not protect MTs from cold-induced depolymerization. Pre-polymerized MTs were incubated in the presence of 10 µM of kinase inhibitors, 10 µM paclitaxel or DMSO for 30 min at 37°C and then incubated at 4°C for 30 min. Free depolymerized tubulin (supernatant, S) and MTs (pellet, P) were separated by centrifugation on a sucrose cushion, as described in the *Materials and Methods* section. Equal volumes of the S and P fractions were subjected to SDS-PAGE separation and proteins were stained by coomassie blue.

To test whether the compounds directly interact with tubulin, we examined their ability to interfere with *in vitro* pure tubulin self-assembly. The well-characterized MT-stabilizing agent PTX can induce MT assembly *in vitro* at low tubulin concentrations that would not induce spontaneous MT assembly (Tang et al., 2017). We found that, unlike PTX, none of the compounds were able to induce MT assembly *in vitro* (**Figure 5A**). We also wondered if the compounds could stabilize preformed MTs by binding to the MT lattice for instance. To test this hypothesis, we prepared purified MTs that were subsequently incubated with the compounds before assaying their stability to cold-induced depolymerization. At the end of this experiment, stable MTs were separated from depolymerized tubulin by a centrifugation through a warm sucrose cushion. As shown in **Figure 5B**, none of the compounds was able to stabilize MTs, indicating that they do not directly bind to tubulin, in contrast to PTX.

**Figure 6:**
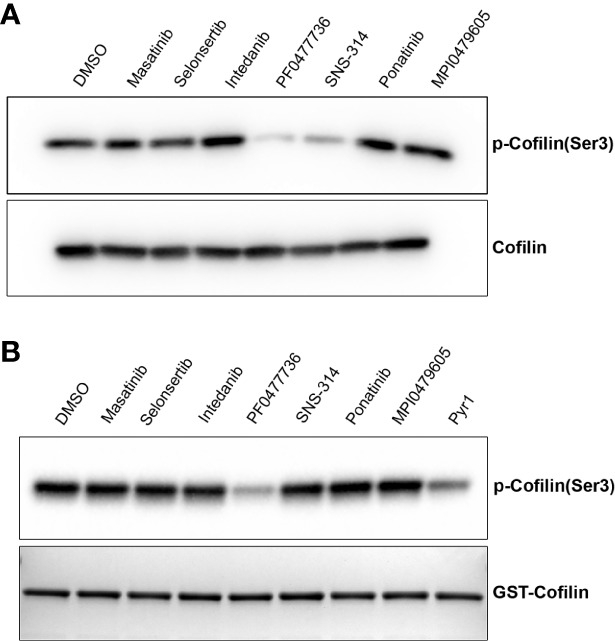
Some kinase inhibitors target the cofilin pathway. **(A)** Cellular LIMK activity is reduced when cells are treated with some kinase inhibitors. HeLa cells were treated for 2 h with 10 µM of kinase inhibitors. Cell lysates were separated by SDS-PAGE and immunoblotted using anti-phosphocofilin (ser3) or anti-cofilin antibodies as indicated. **(B)** *In vitro* phosphorylation of recombinant GST-cofilin. GST-cofilin (2.9 µM) was incubated with LIMK (100 nM) in kinase buffer in the presence of the different kinase inhibitors as described in the *Materials and Methods* section. DMSO was used as negative control. Proteins were separated by SDS-PAGE and cofilin phosphorylation was analyzed after immunoblotting using the anti-phosphocofilin (ser3) antibody. A coomassie-stained SDS-PAGE gel shows the corresponding content of GST-cofilin in each sample.

LIM kinases (LIMK) are among the kinases reported to control MT dynamics during the interphase of the cell cycle. Upon their inhibition, a stabilization of the interphase MT network has been described (Gorovoy et al., 2005; Prudent et al., 2012; Mardilovich et al., 2015; Prunier et al., 2016). We thus assayed the effect of the compounds on cellular LIMK activity by analyzing the phosphorylation of its major substrate cofilin. We found that upon cell treatment with SNS-314 mesylate and PF0477736, the intracellular cofilin was less phosphorylated, indicating that these compounds likely target upstream components of the pathway leading to cofilin phosphorylation (**Figure 6A**). An *in vitro* analysis of the direct inhibitory effect of the compounds on recombinant LIMK activity, using also recombinant cofilin as substrate (**Figure 6B**) showed that PF0477736 is indeed able to directly inhibit LIMK. PF0477736 was originally described as a Chk1/Chk2 inhibitor. The MT stabilizing effect of this compound could thus be ascribed to a combination of its inhibitory action on LIMK and on Chk1/Chk2, which have been shown to be able to phosphorylate and inactivate the MT-associated protein Tau (Mendoza et al., 2013).

SNS-414 mesylate is a potent, but non-selective Aurora Kinase inhibitor. It has been reported that Aurora Kinase A—but not Aurora Kinase B—inhibition, can control MT dynamics in interphase cells (Lorenzo et al., 2009). Thus the MT stabilization that we observed when cells were treated with this compound could result from its inhibitory effect on Aurora Kinase A in conjunction with its indirect inhibitory effect on LIMK activity. Indeed a positive regulation of the LIMK2 isoform by Aurora A has been described, that could explain this indirect effect (Johnson et al., 2012).

For the other most potent selected kinase inhibitors, i.e. selonsertib, intedanib, and masatinib, we conducted additional experiments, using other known available inhibitors of these kinases with different scaffolds, to investigate if the MT stabilizing effect is due to the inhibition of the targeted kinase, or an off-target effect of these compounds.

**Figure 7:**
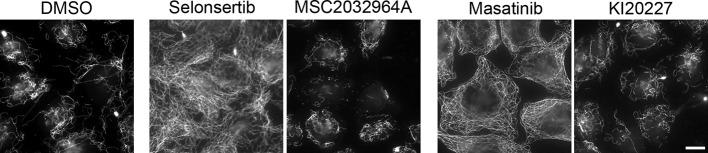
Comparative analysis of the effect of structurally different inhibitors of ASK1 (Selonsertib and MSC203296A) and of tyrosine-kinase receptors (Masatinib and KI20227) on microtubule stability, as assessed by the resistance of the microtubule network to a CA4-induced depolymerization. HeLa cells were incubated for 90 min with 0.25% DMSO (control) or 10 µM of the indicated kinase inhibitors. CA4 (0.5 µM) was then added for 30 min. Cells were permeabilized and processed for immunofluorescence using the anti-α-tubulin antibody. Scale bar, 10 µm.

Selonsertib is an apoptosis signal-regulating kinase 1 (ASK1) inhibitor (Loomba et al., 2018). Among the different described functions of ASK1, its involvement in cell division has been reported, by regulating spindle orientation and positioning through phosphorylation of the microtubule-binding protein EB1 (Luo et al., 2016). However, no ASK1-dependent regulation of interphase MT dynamics has been described, to our knowledge. In order to determine if the selonsertib-induced stabilization of the MT network is due to ASK1 inhibition, we tested the MT stabilizing effect of a structurally unrelated ASK1 inhibitor, MSC2032964A (Guo et al., 2010). We found that MSC2032964A does not induce a MT resistance to CA4 induced depolymerization (**Figure 7**), indicating that the interphase MT stabilization by selonsertib treatment is most probably not due to ASK1 inhibition.

Masatinib and intedanib (also known as nintedanib or BIBF 1120) are inhibitors of tyrosine kinase receptors. They both inhibit platelet-derived growth factor receptor (PDGFR) (Dubreuil et al., 2009; Papadopoulos and Lennartsson, 2018) and fibroblast growth factor receptor (FGFR) (Papadopoulos and Lennartsson, 2018). In addition, masatinib targets c-Kit and the colony stimulating factor-1 receptor (CSF-1R) whereas intedanib targets the vascular endothelial growth factor receptor (VEGFR) and FLT3. Interestingly, imatinib, another tyrosine kinase receptor inhibitor, that targets BCR-Abl as well as cKIT or CSF-1R, was present in the library but not selected as a hit in the initial screen. Similarly, ponatinib, which also inhibits BCR-Abl, cKit, PDGFR, FGFR, VEGFR, and Flt3 presented only a moderate MT stabilizing activity (**Figure 4A**). We also tested KI20227, an inhibitor that targets CSF-1R, VEGFR, c-Kit, and PDGFR, and we observed no significant effect on MT stability as assessed by the resistance of cellular MTs to a CA4-induced depolymerization (**Figure 7**). Altogether, these data suggest that the observed MT stabilizing effect is due to an off-target effect of masatinib and intedanib.

## Discussion

A full awareness of a drug’s mechanism of action is a challenge in the development of target-specific drugs with improved safety profiles. Non-kinase targets of protein kinase inhibitors are regularly reported (Munoz, 2017). Several kinase inhibitors have been reported to bind tubulin and inhibit its polymerization (Aoyama et al., 2014; Bohnacker et al., 2017; Steinmetz and Prota, 2018). Their effect on tubulin polymerization, rather than on kinase activity, is often responsible for their anticancer activity (Munoz, 2017). Kinase inhibitors targeting tubulin cannot be recognized in the commonly used kinome-wide screens. Moreover, as both kinase and tubulin anticancer drugs promote cell-cycle arrest and have antiproliferative activities, it is not easy to identify their exact modes of action by overall toxicity and cell cycle assays.

So far, besides the LIMK inhibitor Pyr1 (Prudent et al., 2012), no kinase inhibitor capable of stabilizing the MT network has been described. However, as a stabilization of the MT network must be sought to be seen, the stabilizing effect of inhibitory compounds can remain completely unnoticed if it has not been specifically looked for it.

Here, we have screened a library of kinase inhibitors for their ability to stabilize the MT network. Interestingly, when tested at 10 µM, only seven compounds out of 190 compounds of the original library were found positive, yielding a 3.7% hit rate similar to commonly reported HTS hit rates. The stabilizing activity was confirmed using two other independent assays: the MT detyrosination status and, for the most potent compounds, the analysis of the modification of MT dynamic behavior using videomicroscopy. These assays report different aspects of MT dynamics and their regulation and may give different results. For instance, enhanced MT detyrosination can be the indirect result of an enhanced microtubule stability (Lafanechere and Job, 2000) as well as an activation of the enzyme that catalyzes tubulin detyrosination (Aillaud et al., 2017) or an inhibition of the enzyme that catalyzes tubulin re-tyrosination (Ersfeld et al., 1993). Moreover, the sensibility of these assays is different. The quantitative analysis of the MT dynamics using videomicroscopy analysis is more sensitive, but less parallelizable. Nevertheless, all these different assays give convergent results, which reinforces the finding of the MT stabilizing activity of the compounds with masatinib, selonsertib, intedanib, and SNS-314 mesylate being the most active drugs.

In cell-free systems, these compounds have been reported to inhibit their targeted kinase when applied in the nanomolar range. However, often higher concentrations are necessary to observe their effect on target dependent cellular processes, like apoptosis, inhibition of proliferation, etc. [1–30 µM for selonsertib (Ji et al., 2019; Mukherjee et al., 2019), 0.1–1 µM for intedanib (Lehtonen et al., 2016; Knüppel et al., 2017), 0.1–10 µM for masatinib (Dubreuil et al., 2009; Trias et al., 2016), 1–10 nM for SNS-314 mesylate (Oslob et al., 2008)]. Our results show that the stabilizing effect on cellular MTs is observed when compounds are used in the 5–10 µM range, i.e. often at similar concentrations than those previously reported to obtain a cellular effect. This indicates that the doses necessary to inhibit these kinases in cells may cause a stabilization of MTs.

Moreover, the intracellular concentration of the compounds depends on the duration of exposure of the cell to the compounds, of the equilibrium between the entry and exit of the compounds through the plasma membrane, and of their metabolism within the cell. We have shown here that longer exposure of cells to lower doses of some of these compounds can also induce a stabilization of the MTs. This is to be taken into account, especially for those of these inhibitors, which are tested or used clinically and therefore often delivered at repeated doses and which may reach high intracellular concentrations.

Among the hits we have found, the effect of some compounds on MTs can be explained directly by the inhibition of the targeted kinase. This is the case for instance for MPI047960, an inhibitor of the Monopolar spindle 1 (Mps1) kinase, whose activity plays a primary role in the mitotic checkpoint (Abrieu et al., 2001) by regulating the kinetochore-microtubule attachment stability (Maciejowski et al., 2017). Mps1 kinase is also reported to reside predominantly in the cytoplasm where it is an important MT interphase regulator (Mattison et al., 2011; Jia et al., 2015). Moreover, it has been shown that Mps1 kinase can bind to MTs *in vitro* (Stucke et al., 2004). Thus, an attractive explanation of the observed MPI047960 partial stabilizing effect on interphase MTs is that it is consecutive to Mps1 kinase inhibition.

We have also found that the MT stabilizing effect of two of the selected compounds, SNS-314 mesylate and PF0477736, whose primary targets are Aurora Kinase and Chk1/Chk2, respectively, could be due to their inhibitory activity on LIMK, which has not been previously described. The important MT stabilizing effect that we have observed for SNS-314 mesylate can be the source of the *in vivo* synergy with paclitaxel that has been described for this compound in a colon carcinoma model (VanderPorten et al., 2009).

Surprisingly, we have discovered a strong MT stabilizing activity for selonsertib, masatinib, and intedanib and a moderate one for ponatinib.

Selonsertib is currently evaluated in Phase 3 clinical studies for the treatment of non-alcoholic steatohepatitis (https://www.clinicaltrials.gov). The rationale is that ASK1 inhibition can reduce hepatic inflammation, apoptosis, and fibrosis through inhibition of stress response pathways. Some reported side effects such as numbness of the extremities (Loomba et al., 2018) suggest the occurrence of peripheral neuropathies. Similar undesirable side effects have been reported in patients treated with taxanes. Thus, in the light of our observations, these adverse effects of selonsertib could result from its effect on the MT network.

Masatinib is currently sold as a veterinary drug (Masivet^®^) to treat canine mastocytosis with mutated cKit receptors. Because it inhibits enzymes involved in inflammation, and as nerve inflammation is a significant aspect of amyotrophic lateral sclerosis (ALS), masatinib is also promising for the treatment of ALS (Scott, 2017; Trias et al., 2018). A phase 2/3 study to compare the efficacy and safety of masatinib with other existing ALS treatments has been completed with encouraging positive results (Mora et al., 2019). In oncology, masatinib is currently tested for the treatment of pancreas and prostate cancer and the results of these two phase 3 trials are expected in 2020. Knowledge of the unexpected effects of masatinib on MTs may be useful in interpreting the results of these studies, as well as possible side effects and to design further best drug combinations.

Intedanib is clinically indicated to treat idiopathic pulmonary fibrosis. It is also currently approved for use in combination with docetaxel for treating advanced lung cancer that has progressed after first-line chemotherapy. Indeed, in a phase 3 study, progression free survival was found significantly improved in the docetaxel plus intedanib group compared with the docetaxel plus placebo group (Reck et al., 2014). In addition to the original rationale for combining the anti-angiogenic effect of intedanib, which is due to its inhibitory effect on VEGFR, with the anti-tumor effect of docetaxel, our finding provides new mechanistic insights. Indeed, the underlying mechanism could be an additive effect of two microtubule-stabilizing compounds. Moreover, when administrated alone (Ofev^®^, Vargatef^®^), intedanib induces frequently described adverse effects such as neutropenia or peripheral neuropathies that could be ascribed to its effect on MTs.

Ponatinib is clinically approved for the treatment of chronic myeloid leukemia and Philadelphia chromosome-positive acute lymphoblastic leukemia, specifically targeting the *BCR-ABL* gene mutation, T315I (Tan et al., 2019). Ponatinib is also pre-clinically evaluated in other tumor types, with encouraging results. Our data encourage a re-analysis of the tumor cell effect of ponatinib, with a focus on its effect on the MT network.

Overall, our results show that several kinase inhibitors, initially thought to exert their therapeutic effect through a defined mechanism on the diverse kinase pathways can also behave as more general cytotoxic drugs, due to the alteration of MT dynamics. In light of these data, a possible repurposing of some of these drugs is conceivable.

Finally, although the binding site of taxanes has some structural characteristics similar to those of the kinase catalytic site (Steinmetz and Prota, 2018), none of the inhibitors that our assay has selected directly binds to tubulin. Understanding precisely how these inhibitors induce MT stabilization will allow the identification of new regulators of MT dynamics. This is of interest not only in terms of knowledge, but also for the identification of new targets for anticancer therapies.

## Data Availability Statement

All datasets generated for this study are included in the article/**Supplementary Material**.

## Author Contributions

SR-R and LP designed, performed, and analyzed the experiments characterizing the stabilizing effect of the selected compounds and searched for the compound mechanism of action. SM and CR set up and miniaturized the assay and conducted dose-effect analysis of the selected compounds. CB automatized the assay and screened the kinase inhibitor chemical library. YF synthesized and provided LIM Kinase inhibitors. M-OF, AA, KS, and LL supervised the research. LL directed the project and wrote the manuscript. All authors provided critical analysis of the manuscript.

## Funding

This work was supported by INSERM, Université Grenoble Alpes, CNRS, and by l'Institut National du Cancer (INCa, PLBIO16186), and Association Le Cancer du Sein- Parlons-en, to LL; by the LabEx GRAL (ANR-10-LABX-49-01), supported by the University Grenoble Alpes graduate school (ANR-17-EURE-0003) to M-OF.

## Conflict of Interest

Author YF was employed by company Reaction Biology Corporation.

The remaining authors declare that the research was conducted in the absence of any commercial or financial relationships that could be construed as a potential conflict of interest.

## Supplementary Material

Supplementary Movie 1 Time-lapse microscopy was performed using cells infected with the mRuby3-MT+tips construct and treated for 2 h with DMSO (control), 20 µM selonsertib, intedanib, and SNS-314 mesylate or 10 µM of masatinib, as indicated. Images were collected every 1.5 sec for 2 min and films are 11× accelerated.
